# Full-Genome Sequences of Two Newcastle Disease Virus Strains Isolated in West Java, Indonesia

**DOI:** 10.1128/MRA.00221-20

**Published:** 2020-06-11

**Authors:** P. Pandarangga, M. I. Cahyono, M. M. McAllister, A. E. Peaston, R. Tearle, W. Y. Low, P. T. K. Doan, M. Rabiei, J. Ignjatovic, N. P. I. Dharmayanti, R. Indriani, S. Tarigan, F. Hemmatzadeh

**Affiliations:** a School of Animal and Veterinary Sciences, University of Adelaide, Adelaide, South Australia, Australia; b Department of Veterinary Pathology, Nusa Cendana University, Kupang, Indonesia; c Department of Veterinary Medicine, Tay Nguyen University, Buon Ma Thuot, Dak Lak, Vietnam; d Indonesian Research Centre for Veterinary Science, Bogor, West Java, Indonesia; e School of Veterinary Science, University of Melbourne, Melbourne, Victoria, Australia; f Davies Research Centre, School of Animal and Veterinary Sciences, University of Adelaide, Adelaide, South Australia, Australia; DOE Joint Genome Institute

## Abstract

The full-genome sequences of strains chicken/Indonesia/Cilebut/010WJ/2015 and chicken/Indonesia/ITA/012WJ/1951, isolated in West Java, Indonesia, in 2015 and 1951, respectively, were examined. Chicken/Indonesia/Cilebut/010WJ/2015 (genotype VII) caused a 2015 disease outbreak in Indonesia, and chicken/Indonesia/ITA/012WJ/1951 (genotype VI) is used as a standard strain for challenge in Newcastle disease virus (NDV) vaccine trials.

## ANNOUNCEMENT

In Indonesia, Newcastle disease (ND) outbreaks have caused high mortality in commercial chickens, even when vaccinated with attenuated Newcastle disease virus (NDV) (1). Formally, NDV is *Avian orthoavulavirus 1*, the type virus of the *Orthoavulavirus* genus in the *Paramyxoviridae* family (2). Genotype VII NDV predominates in Southeast Asia (3, 4), but local vaccines mostly contain genotype II strain LaSota (5). Chicken/Indonesia/Cilebut/010WJ/2015 (Cilebut) and chicken/Indonesia/ITA/012WJ/1951 (ITA) are virulent NDV strains (5). Strain ITA was collected in West Java, Indonesia, in 1951 and today is used in challenge trials to test the efficacy of vaccines (5). Strain Cilebut was collected in 2015 from chicken brains in West Java. We hypothesize that antigenic mismatch between genotype VII and strain ITA that is used in challenge trials has caused unreliability of vaccine challenge test results, enabling ND outbreaks in vaccinated chickens.

Full-genome sequences of strains Cilebut and ITA were analyzed. Both strains were propagated in the allantoic cavities of 9-day-old specific-pathogen-free (SPF) chicken eggs, fluid was collected by day 4, and infections were confirmed using a hemagglutination test (6). Viral RNA was extracted from the allantoic fluid using RNeasy Plus universal kits (Qiagen). The concentration and quality of the viral RNA were measured using the 2200 TapeStation system (Agilent Technologies, USA). cDNA libraries were synthesized using a Kapa stranded RNA library kit and sequenced on an Illumina MiSeq instrument to generate 2 × 300-nucleotide (nt) reads, 989,005 paired reads for strain Cilebut and 636,469 paired reads for strain ITA.

The raw sequence data were assembled using Unicycler V0.4.8 with default parameters (7). The assembled scaffolds were extracted using Bandage V0.8.1 (8). Strain ITA had the following 3 contigs: contig 2 (length, 14,539 bp; coverage, 246.87-fold), contig 160 (length, 7 bp; coverage, 75.91-fold), and contig 152 (length, 823 bp; coverage, 175.8-fold). The strain ITA contigs were compared to existing NDV sequences using BLAST and showed 94 to 96% identity with chicken/U.S.(CA)/1083(Fontana)/72 (Fontana; GenBank accession number AY562988.1). These contigs were also aligned to strain Fontana using minimap2 V2.17 (9) to determine their orientation and order; the 3 contigs aligned contiguously and without gap or ambiguity to reference sequence of strain Fontana as follows: contig 152, from 102 to 822 bp; contig 160, from 823 to 829 bp; and contig 2, from 830 to 15,369 bp. Hence, a complete genome was reconstructed by concatenating the three contigs in the corresponding order. The final genome of strain ITA had a total length of 15,369 bp, 1,372-fold coverage, and a GC content of 46.0%. The sequence was compared to all known sequences using BLAST with the NCBI nonredundant/nucleotide (nr/nt) databases. The top BLAST hit showed 94.7% identity with strain Fontana. The genome was annotated using Geneious V2020.0.3. Sequence alignment and phylogenetic analysis confirmed that strain ITA belongs to genotype VI with ^112^R-R-Q-K-R-F^117^ as the cleavage site motif of the fusion protein. The contig of strain Cilebut had a total length of 15,225 bp, 246-fold coverage, and a GC content of 46.2%. The top BLAST hit showed 97.07% identity with NDV isolate IBS005/11 (GenBank accession number KR074405.1). Annotation was performed as described above. Sequence alignment and phylogenetic analysis of strain Cilebut revealed that it belongs to genotype VII, with the fusion protein cleavage site motif ^112^R-R-Q-K-R-F^117^.

Genotype VII NDVs are predominantly responsible for ND outbreaks in Indonesia; however, genotype II is used in most Indonesian vaccines, and the standard challenge strain used in vaccine efficacy trials is from genotype VI. Antigenic disparities between the vaccine, challenge, and field strains of NDV may enable disease outbreaks in vaccinated chickens.

### Data availability.

The GenBank and Sequence Read Archive (SRA) accession numbers for chicken/Indonesia/Cilebut/010WJ/2015 are MN727299 and SRR11593163, respectively, and those for chicken/Indonesia/ITA/012WJ/1951 are MN727300 and SRR11593165, respectively.
